# First Evidence of Acyl-Hydrolase/Lipase Activity From Human Probiotic Bacteria: *Lactobacillus rhamnosus* GG and *Bifidobacterium longum* NCC 2705

**DOI:** 10.3389/fmicb.2020.01534

**Published:** 2020-07-24

**Authors:** Panagiotis Manasian, Atma-Sol Bustos, Björn Pålsson, Andreas Håkansson, J. Mauricio Peñarrieta, Lars Nilsson, Javier A. Linares-Pastén

**Affiliations:** ^1^Biotechnology, Faculty of Engineering, Lunds Tekniska Högskola (LTH), Lund University, Lund, Sweden; ^2^Food Technology, Faculty of Engineering, Lunds Tekniska Högskola (LTH), Lund University, Lund, Sweden; ^3^Faculty of Pure and Natural Sciences, School of Chemistry, Universidad Mayor de San Andrés, La Paz, Bolivia

**Keywords:** acyl-hydrolase/lipase, *Lactobacillus rhamnosus* GG, *Biffidobacterium longum*, asymmetrical flow field-flow fractionation, differential scanning fluorimetry

## Abstract

*Lactobacillus rhamnosus* GG (ATCC 53103) and *Bifidobacterium longum* NCC 2705 are among the most studied probiotics. However, the first evidence of acyl hydrolase/lipase of two annotated proteins, one in each genome of these strains, is reported in this work. Signal peptide analysis has predicted that these proteins are exported to the extracellular medium. Both proteins were produced in *Escherichia coli*, purified and characterized. Molecular masses (without signal peptides) were 27 and 52.3 kDa for the proteins of *L. rhamnosus* and *B. longum*, respectivel*y*. Asymmetrical flow field-flow fractionation analysis has shown that both proteins are present as monomers in their native forms at pH 7. Both have shown enzymatic activity on *p*NP-laurate at pH 7 and 37°C. The enzyme from *L. rhamnosus* was characterized deeper, showing preference on *p*NP-esters with short chain fatty acids. In addition, a computational model of the 3D structure has allowed the prediction of the catalytic amino acids. The enzymatic activities using synthetic substrates were very low for both enzymes. The investigation of natural substrates and biological functions of these enzymes is still open.

## Introduction

Probiotics are microorganisms that provide beneficial effects for health and are gaining great interest both in the prevention as well as in the treatment of different diseases. The WHO (FAO/WHO) defines probiotic as “live microorganisms that, when administered in adequate amounts, confer a health benefit on the host” (Hill et al., 2014). *Lactobacillus rhamnosus* GG (ATCC 53103) and *Bifidobacterium longum* NCC 2705 are among the most studied probiotics. Several evidences support the positive effect of these strains against obesity, diabetes, inflammatory bowel disease, cancer, mental disorders, infections, diarrhea, immune disorders, systemic inflammation and others (Brusaferro et al., 2018; Consortium, 2018; Capurso, 2019). Indeed, a recent search in Google Patents gave more than 1250 and 1050 patents related with *B. longum* NCC 2705 and *L. rhamnosus* GG, respectively. In addition, *L. rhamnosus* is one of the most used bacteria in the probiotic food industry, which includes the manufacture of milk-based products such as yogurt-type drinks, but also, probiotic fruit juices, berry soups and products based in fermented cereals and soy (Saxelin, 2008).

*L. rhamnosus* GG (ATCC 53103) is Gram positive anaerobic bacteria that inhabit in the gastrointestinal tract (GIT) but could also be recovered from oral cavity, tonsils, and vagina (Segers and Lebeer, 2014). *L. rhamnosus* GG (ATCC 53103) was isolated from the gastrointestinal tract in 1983 (Gorbach and Goldin, 1989) and its genome sequenced in 2009 (Kankainen et al., 2009). It is a circular chromosome of 300,5051 pb with 2834 predicted protein-coding genes. *B. longum* is a non-halophilic, anaerobic, Gram positive bacteria commonly present in the animal and human intestines (Consortium, 2018). It is associated with the gastrointestinal tract and promotes several health benefits, including reduction of irritable bowel syndrome (Brigidi et al., 2001), improves the immune response in infants (Young et al., 2004) and also helps to maintain homeostasis in the intestinal ecosystem promoting normal digestion (Consortium, 2018). *B. longum* NCC 2705 contains a 2,256,646 bp genome organized in a circular chromosome (Schell et al., 2002). Most of the studies at enzymatic level are focused on proteins involved in the metabolism of carbohydrates and amino acids, transport systems and defense mechanism. Indeed, the genomes of *L. rhamnosus* and *B. longum* have shown a relatively high number of these type of putative proteins comparing with other intestinal lactobacilli (Schell et al., 2002; Kankainen et al., 2009).

Despite the number of studies on *L. rhamnosus* and *B. longum*, to the best of our knowledge, there is lack of reports on lipolytic activity in these probiotic strains. Lipolytic enzymes include carboxylesterases and lipases, which are classified into 19 families based on phylogenetic criteria, conserved motifs and biological function (Kovacic et al., 2019). The metabolism of lipids, including esters of large and short chain fatty acids, in the gastrointestinal tract plays an important role in several cellular functions associated with disorders, such as obesity and pathological conditions. Thus, the study of lipolytic enzymes secreted by gastrointestinal microorganisms, including probiotics, could contribute to understand the potential effects of these strains in the metabolism of lipids in the intestinal tract and their health implications. The genome analysis of *L. rhamnosus* GG and *B. longum* NCC 2705 has shown two putative lipase/acyl-hydrolases containing signal peptides in the N-terminal, one in each strain, which indicates that these proteins can be secreted to the extracellular medium. Therefore, the purpose of this work was to investigate the activity of these proteins, named *Lr*Lyp and *B*lLyp (from *L. rhamnosus* and *B. longum*, respectively).

*Lr*Lyp and *Bl*Lyp were produced in *Escherichia coli* and their enzymatic and biophysical properties were studied. Substrate selectivity and activity were evaluated with series of synthetic *para*-nitrophenyl esters, while apparent kinetic constants were obtained at pH 7 and 37°C, using *para*-nitrophenyl laurate (*p*NP-laurate) as substrate. This synthetic ester has a 12-carbon atom chain, corresponding to a medium-size fatty acid, and it is widely used for characterizing lipase activity from different sources, including bacteria (Castro-Ochoa et al., 2005). The biophysical characterization was focused on the determination of: (1) optimal temperature; (2) protein stability, in terms of melting temperature determined by differential scanning fluorimetry (DSF) in a range of pH from 4 to 10; and (3) multimerization, studied by asymmetrical flow field-flow fractionation (AF4) coupled to multiangle light scattering (MALS). AF4 separates multimeric proteins and aggregates based on their diffusion coefficient and, compared to size exclusion chromatography (SEC), does not have a stationary phase which reduce the potential loss of analytes from adsorption or shear-induced degradation (Wahlund and Nilsson, 2012; Choi et al., 2018).

Finally, there are no structures with significant homology to *Lr*Lyp neither *Bl*Lyp in the protein data bank (PDB) (last search on 2019.10.27). This has limited the construction of a reliable computational 3D model for *Bl*Lyp. Nevertheless, a model for *Lr*Lyp was obtained using remote homology modeling techniques, allowing the prediction of the catalytic residues among other structural features.

The biological function and natural substrates of *Lr*Lyp and *Bl*Lyp remains unknown. However, this work contributes with the first evidences of acyl hydrolase/lipase activity for these enzymes encoded by the genomes of *Lactobacillus rhamnosus* GG (ATCC 53103) and *Bifidobacterium longum* NCC 2705, respectively.

## Materials and Methods

### Genes Selection and Synthesis

Annotated esterases and lipases from the genomes of *Lactobacillus rhamnosus* GG (ATCC 53103) (GenBanck access code: NC_013198.1) and *Bifidobacterium longum* NCC 2705 (NC_004307) were subjected to signal peptide analysis using SignalP 4.0 Server (Petersen et al., 2011). Loci LGG_RS06710 (*L. rhamnosus*) and BL1109 (*B. longum*) were synthesized (GenScript, United States) with codon optimized for *Escherichia coli*, excluding the region encoding the signal peptides. These genes were cloned in a pET21b(+) vector between the *Nde*I and *Xho*I restriction sites. A 6-histidine tag encoded by the vector backbone was added in the C-terminal of each gene. The resulting plasmids were named pET21b:LGG_RS06710 and pET21b:BL1109.

### Bioinformatic Analysis

Motifs around the catalytic serine of different families of lipolytic enzymes (Kovacic et al., 2019) were searched in the aminoacidic sequences of *Lr*Lyp and *Bl*Lyp in order to determine their classification. Other conserved motifs were determined by multiple sequence alignments with selected characterized enzymes. The alignments were performed using the Clustal Omega (1.2.4) server.^1^

### Production of Recombinant Proteins

Different strains of *E. coli*, including BL21(DE3), Origami2, and Artic Express, were transformed with the plasmids pET21b:LGG_RS06710 and pET21b:BL1109. Each plasmid was introduced into chemically competent cells (Novagen, United States) by thermic shock (4°C 10 min, 42°C 2 min, 4°C 10 min). All cells were grown in LB medium supplemented with 100 μg/mL of ampicillin, additional 20 μg/mL gentamicin was added in the cultivation media of *E. coli* Artic Express. All cultivations were incubated in shake flasks (with agitation) at 37°C until reach an OD_λ = 600 nm_ of 0.6. Then, 1 mM IPTG was added and the incubation temperature for the recombinants *E. coli* BL21(DE3) and Origami2 was decreased to 30°C for 12 h, while for the recombinant *E. coli* Artic Express was decreased to 8°C for 24 h.

### Protein Purification

Cell pellets were harvested by centrifugation at 4500 g for 20 min and resuspended in 1/10 volume of binding buffer (100 mM tris, NaCl 500 mM adjusted to pH 7.4 with HCl). Thereafter, the cell suspensions were sonicated for 5 min and centrifuged at 14,000 g. The precipitates were discarded, and the supernatants were subjected to SDS-PAGE analysis in order to determine the level of expression of the recombinant proteins. Protein purification was performed by IMAC, each supernatant was injected in a 5-mL HisTrap column FF (GE Healthcare, Uppsala, Sweden) previously equilibrated with binding buffer. Next, the column was washed with 15 column volumes of binding buffer. The recombinant protein was eluted with elution buffer pH 7.4 (50 mM sodium phosphate, 0.5 M NaCl, and 0.5 M imidazole). Finally, imidazole was removed by dialysis. Protein purity was determined by SDS-PAGE. Second purifications, using a 5-mL HisTrap column HP (GE Healthcare, Uppsala, Sweden), were performed when needed. The concentration of pure proteins was quantified spectrophotometrically at 280 nm.

### Determination of Stability by Differential Scanning Fluorometry

Thermal stability of the enzyme in different pH values was determined using the Prometheus NT 48 nanoDSF (NanoTemper Technologies, GmbH, Munich Germany) (Choi et al., 2018). Enzyme samples at 0.5 g/L were prepared in pH 4.3, 5.3, 6.2, 7, 8 (McIlvaine buffer system, 100 mM), 9 and 9.6 (glycin-NaOH buffer, 100 mM). Then, 10 μL of every sample was directly loaded in the instrument capillaries. Intrinsic fluorescence at emission wavelengths of 330 and 350 nm was monitored in a temperature gradient from 20 to 90°C with a temperature ramp of 1°C per min. All data analysis was performed using the PR control software (Version 2.0, Munich Germany).

### Substrate Selectivity

Stock solutions of the following *para*-nitrophenyl esters: *p*NP-acetate (C2), *p*NP-butyrate (C4), *p*NP-caprylate (C8), *p*NP-laurate (C12), *p*NP-myristate (C14), *p*NP-palmitate (C16), and *p*NP-stearate (C18), were prepared in concentrations of 200 (C2,C4,C8), 100 (C12, C14), and 50 (C16, C18) mM. A mixture of acetonitrile and 2-propanol (1:1) was used as solvent. The mixtures were subjected to ultrasound bath until a clear solution was obtained. All substrates and solvents were purchased from Sigma Aldrich (Darmstadt, Germany). The reactions were prepared in volumes of 210 μL, containing 50 mM phosphate buffer pH 7.2, 1 mM substrate and 0.074 g/L of enzyme. The reactions started by adding the enzyme and monitored at 410 nm of wavelength in a microplate spectrophotometer (Thermo Scientific^TM^, Multiskan^TM^ GO) during 30 min at 37°C.

### Determination of Optimal Temperature

The optimal temperature was determined for *Lr*Lyp in a range from 20 to 60°C. The pH was set to 7.2 with 20 mM McIlvain buffers and 5 mM of *p*-nitrophenyl laurate was used as substrate. The reactions were initiated by adding the enzyme (0.133 g/L) and stopped after 40 min by heating at 94°C for 10 min and cooling down to 4°C. All reactions were performed in triplicates and using controls, which consisted in same reactions mixtures, except enzyme. All reactions, including controls, were incubated in thermocycler (Biometra T-gradient from AnalyticJena, Germany). The formed product, *p*-nitrophenol, was quantified in a microplate spectrophotometer (Thermo Scientific^TM^, Multiskan^TM^ GO) at 410 nm.

### Determination of Kinetic Constants

A stock solution of *p*NP-laurate, was prepared in a concentration of 10 mM, dissolving *para*-nitrophenyl laurate flakes in a mixture of water, DMSO and triton-X 100 (2:1:1, respectively) (modified from Mastihuba et al., 2002). The mixture was subjected to ultrasonic bath at 50°C for 10 min until an oily clear solution was obtained. Initial rates were determined in a range of 0.1–9 mM *p*-nitrophenyl laurate. Reactions were initiated by adding the respective enzyme at concentrations of 0.133 g/L of *Lr*Lyp and 0.358g/L of *Bl*Lyp. The pH 7 was set with 20 mM McIlvaine buffer. The reactions were monitored quantifying the nitrophenol formed along the time, using a microplate spectrophotometer (Thermo Scientific^TM^, Multiskan^TM^ GO) at 410 nm. All reactions were performed by triplicates and using controls, which were prepared with the same composition of reaction mixtures, except enzyme. The correlation between initial rates and substrate concentration was plotted and adjusted to the Michaelis-Menten (Eq. 1) or substrate inhibition kinetics (Eq. 2) model, by non-linear regression methods by the generalized reduced gradient non-linear algorithm, used as implemented in the Solver of Microsoft Excel:

(1) $$v_{0}=\frac{V_{m\,a\,x}^{a\,p\,p}\,\left[S\right]}{K_{M}^{a\,p\,p}+\left[S\right]}$$

(2) $$v_{0}=\frac{V_{m\,a\,x}^{a\,p\,p}\,\left[S\right]}{K_{M}^{a\,p\,p}+\left[S\right]\,\left(1+\frac{\left[S\right]}{K_{I}^{a\,p\,p}}\right)}$$

where *v*_0_ is the initial rate, $V_{m\,a\,x}^{a\,p\,p}$. is the apparent maximum velocity, [*S*] is substrate concentration, $K_{M}^{a\,p\,p}$. is the apparent Michaelis Menten constant and $K_{I}^{a\,p\,p}$. is the apparent dissociation constant for binding.

### Stability

The stability of the enzyme *Lr*Lyp, at 37°C and pH 7, was monitored for 35 days. Residual activities were determined every day the first 5 days, after, once every week. Reactions were performed in triplicates, using 5 mM *p*-nitrophenyl laurate as substrate and quantifying the production of *p*-nitrophenol spectrophotometrically, such as is described before.

### Molecular Modeling

A model of the three-dimensional structure of *Lr*Lyp was obtained from the amino acid sequence, excluding the N-terminal signal peptide. Remote homology recognition techniques were applied using the Phyre2 Protein Fold Recognition Server (Kelley et al., 2015). The refinement of the model was performed by molecular dynamic simulations (MD) of 500 ps (Krieger et al., 2004). This protocol generates snapshots every 25 ps (in format pdb) with a table of energy to identify the best snapshot. The force field used was YAMBER2, implemented in the program YASARA (Krieger and Vriend, 2014). The MD conditions were pH 7.4, temperature 280°K and density 0.997. Additional validation of the model was analyzed using ProSA-web (Wiederstein and Sippl, 2007).

### AF4 Instrumentation

The instrumentation consisted in an Eclipse 3 + Separation System coupled to a Dawn Heleos II multi-angle light scattering (MALS) detector (Wyatt Technology, Dernbach, Germany) with a wavelength of 663.8 nm and to a UV detector (Jasco UV-975 detector, Corp., Tokyo, Japan) with a wavelength of 280 nm. An Agilent 1100 series pump coupled to a vacuum degasser (Agilent Technologies, Waldbronn, Germany was used to deliver the carrier liquid. For the sample injection into the channel, the system was equipped with an auto sampler Agilent 1100 series (Agilent Technologies). The channel used was a trapezoidal long channel (Wyatt Technology) with length of 26.0 cm and outlet inlet widths of 0.6 cm and 2.15 cm, respectively and 350 μm of nominal thickness. A membrane of regenerated cellulose (RC), cut-off of 10 kDa, was used as the accumulation wall (Merck Millipore, Bedford, MA, United States).

### AF4 Sample Preparation

The samples were produced and purified as described in previous sections, resulting in a final concentration of 0.88 mg/mL for *Bl*Lyp and 1.2 mg/mL for *Lr*Lyp. Before the injection, the samples were centrifuged at 11,000 g for 15 min at 4°C. Bovine serum albumin (BSA) at concentration of 2 mg/mL was used as standard to check the performance of the AF4 system and to normalize de MALS detector.

### AF4 Parameters

First, the channel was flushed in elution and focus mode, 1 min each. After that, 100 μL of sample was injected onto the channel at 0.2 mL/min for 2 min. Once the sample was injected (0.088 mg of *Bl*Lyp and 0.12 mg of *Lr*Lyp), 3 min of focus mode were applied before the elution step. A constant cross flow of 5 mL/min was used during the elution period for 15 min, followed by 15 min without cross flow to remove any remnant material. The detector flow was always 1 mL/min. The parameters previously described were used with 5 different carrier liquids: 20 mM of phosphate-citrate buffer at pH 7, 6, 5, 4, and 3 in both lipase samples. A new channel was assembled for every lipase.

### AF4 Data Processing

Astra software 6.1 (Wyatt Technology Europe) was used for the data analysis. For the molecular weight (Mw) of *Bl*Lyp, the value was determined at pH 6 using 13 scattering angles, from 42.8° to 163.3°. The calculations were performed with 1st order Zimm model (Zimm, 1948). The dn/dc value used was 0.185 mL/mg and the UV extinction coefficient 0.863 mL/(mg cm). The second virial coefficient was considered negligible. For both lipases, the hydrodynamic radius (r_h_) was estimated by the Stokes-Einstein equation using FFhydRad 2.1-MATLAB Apps (Håkansson et al., 2012) for the calculations. The channel thickness (*w*) for *Lr*Lyp and for *Bl*Lyp was determined using BSA as a standard.

## Results

### Production of Recombinant *Lr*Lyp and *Bl*Lyp

**FIGURE 1:**
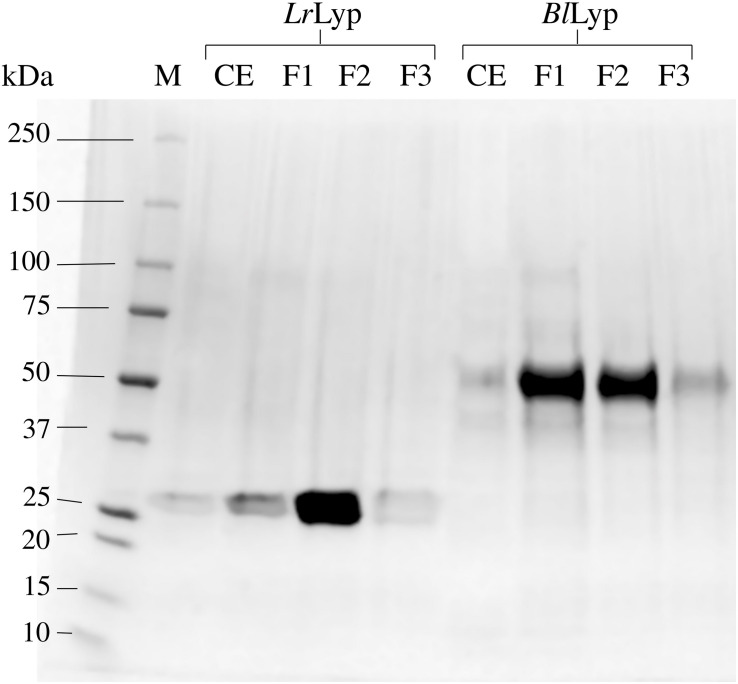
Production and purification of the recombinant *Lr*Lyp (locus LGG_RS06710) and *Bl*Lyp, without signal peptides. SDS-PAGE is showing the molecular weight marker M (Lane 1), the cell extracts (CE) and the different chromatographic fractions (F1–F3) corresponding to each purified protein: *Lr*Lyp (Lanes 3–5) and *Bl*Lyp (Lanes 7–9).

Putative extracellular lipases were searched in the genomes of *Lactobacillus rhamnosus*, strain GG (ATCC 53103) and *Bifidobacterium longum* NCC 2705. Only one locus of a putative annotated lipase/acyl-hydrolase was found in each genome, LGG_RS06710 in *L. rhamnosus*, and BL1109 in *B. longum*. LGG_RS06710 encodes a 281 amino acids protein of 30728.20 Da including a predicted N-terminal signal peptide of 34-amino acids. BL1109 encodes a 480 amino acids protein of 51163.93 Da with 34-amino acids in the N-terminal predicted as signal peptide. Therefore, it is predicted that both proteins are exported to the extracellular medium. The genes encoding these proteins, excluding signal peptides, were synthesized with codons optimized for *Escherichia coli* and successfully produced in strains BL21(DE3), ArticExpres and Origami2. However, the last gave the highest fraction of soluble recombinant protein, estimated in 80% according to the SDS-PAGE analysis. The theoretical molecular weights of these truncated forms (without signal peptide) are 27058.74 Da (*Lr*Lyp) and 52360.25 Da (*Bl*Lyp) from *L. rhamnosus* and *B. longum*, respectively; which is consistent with the molecular mass determined by SDS-PAGE (Figure 1).

### Bioinformatic Analysis

**FIGURE 2:**
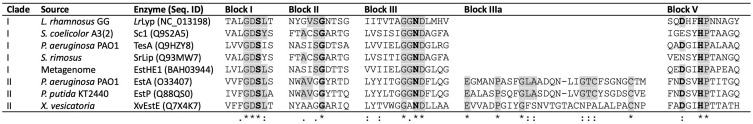
Multiple alignment of conserved blocks in lipolytic enzymes family II. Characterized enzymes were selected for the comparison with *Lr*Lyp from *L. rhamnosus*. Conserved catalytic amino acids are in bolds, S in the block I is the nucleophile, D and H in the block V are the donor proton and catalytic base respectively. G and N in the blocks II and III belong to the oxyanion hole. *Lr*Lyp belongs to clade I, since it lacks of block IIIa (Leščić Ašler et al., 2010). Where (*) denotes conserved or identical amino acid, (:) conservative or amino acid with similar biochemical property, (.) semiconservative and (_) non conservative amino acid.

Amino acid sequence analysis of *Lr*Lyp and *Bl*Lyp has revealed that these enzymes contain conserved motif corresponding to lipolytic enzymes families II and IV respectively. *Lr*Lyp has four blocks: GDSLT (amino acids 53–56), GVSG (aa. 91–94), GGND (aa. 125–128), and HPN (aa. 256–258); as shown in the multiple alignment with other family II enzymes (Figure 2). Previously characterized bacterial GDSL enzymes, including Sc1 from *Streptomyces coelicolor* (Côté and Shareck, 2008), TesA and EstA from *Pseudomonas aeruginosa*, SrLip from *Streptomyces rimosus*, EstP from *Pseudomonas putida* (Leščić Ašler et al., 2010), XvEstE from *Xanthomonas vesicatoriaand* (Talker-Huiber et al., 2003), and EstHE1 obtained from a metagenome (Okamura et al., 2010), were selected for the multiple alignment (Supplementary Information S1).

**FIGURE 3:**
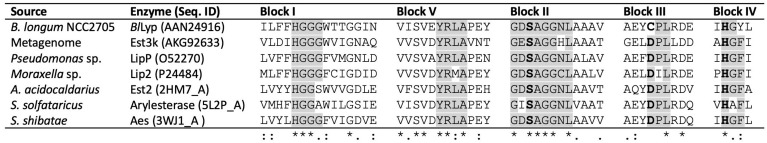
Multiple alignment of conserved blocks in lipolytic enzymes family IV. Characterized enzymes were selected for the comparison with *Bl*Lyp from *B. longum*. Conserved catalytic amino acids are in bolds, S in the block I is the nucleophile, H in block IV is the base, and the proton donor D located in bock III, interestingly is a C in *Bl*Lyp. The recently suggested block V (Kim et al., 2015) containing the consensus sequence YRLA is also present in *Bl*Lyp. Where (*) denotes conserved or identical amino acid, (:) conservative or amino acid with similar biochemical property, (.) semiconservative and (_) non conservative amino acid.

*Bl*Lyp has five conserved motifs present in family IV as shown in Figure 3. The conserved sequences are in five blocks: HGGG (aa. 225–229), YRLA (aa. 257–260), GDSAGGNL (aa. 328–335), CPL (aa. 430–432), and HGI (aa. 460–462). The sequences selected for the multiple alignment correspond to previously characterized family IV lipolytic enzymes, including Est3k obtained from a metagenome (Kim et al., 2015), LipP from *Pseudomonas* sp. (Choo et al., 1998), Lip2 from *Moraxella* sp. (Feller et al., 1991), Est2 from *Alicyclobacillus acidocaldarius* (Mandrich et al., 2008), arylesterase from *Saccharolobus solfataricus* (PDB accession code 5L2P) and Aes from *Saccharolobus shibatae* (Ohara et al., 2014). It is remarkable the presence of cysteine in the motif CPL in *Bl*Lyp, instead of conserved aspartate (DPL) in the rest of the enzymes compared (Supplementary Information S2).

### Optimal pH and Temperature for *Lr*Lyp

**FIGURE 4:**
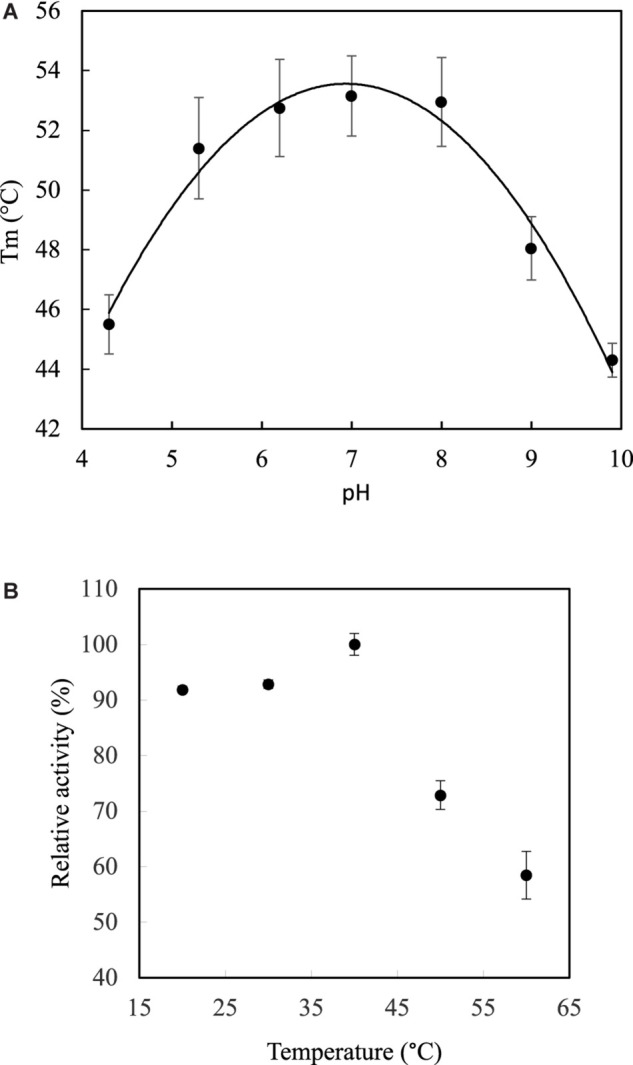
Optimum pH and temperature. **(A)** DSF analysis shows optimum pH between 6 and 8 with a highest transition temperature (melting point Tm) at 53.1°C. **(B)** Temperature curve shows maximum relative activity at 40°C. The pH was 7 and *p*NP-laurate was used as substrate. Error bars denote plus/minus one standard deviation.

Both optimal temperature and pH were determined in terms of thermal stability in a range of pH from 4.3 to 9.9 by DSF. The protein has show the highest stability in a range of pH from 6 to 8 with the optimal melting point of 53.1°C at a pH close to 7 (Figure 4A). Indeed, the data adjusted to the quadratic model *T*_*m*_ = −1.22*pH*^2^ + 16.83*pH*. – 3.19, with a coefficient of determination, *R*^2^, of 98%, which first derivative equal to zero, gave a critical point corresponding to pH 6.88. Therefore, the optimal temperature in terms of relative activity was determined at pH 7, giving maximum activity (100%) in a range from 37 to 40°C (Figure 4B), which is consistent with the physiological temperature. The activity decreases to 90% in a range of 20–30°C, while decreases dramatically below 60% at 60°C.

### Substrate Selectivity for *Lr*Lyp

**FIGURE 5:**
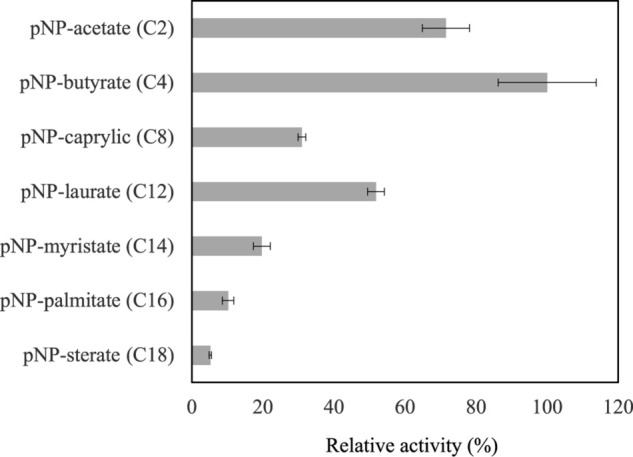
Substrate selectivity for *Lr*Lyp. The reactions were carryout using 1 mM of substrate at 37°C and pH 7.2. Error bars denote plus/minus one standard deviation.

The substrate selectivity was evaluated with seven synthetic *para*-nitrophenyl esters (*p*NP-esters) with linear hydrocarbon chains from C2 to C18. On the other hand, the biodegradable polyester-plastic polycaprolactonate (CAPA 6000) was tested as potential substrate and the phospholipase D activity was assessed as well. Activity was detected only on *p*NP-esters. The highest activity was on *p*NP-butyrate while the lowest was on *p*NP-sterate. Thus, these results show that *Lr*Lyp has preference for short hydrocarbon chain *p*NP-esters (Figure 5 and Table 1). No activity was detected on CAPA 6000 neither phospholipase D activity. The activity of *Bl*Lyp is dramatically lower than the *Lr*Lyp when *p*NP-laurate is used as substrate (Table 1).

**TABLE 1:** Enzymatic activity on *p*NP-esters. The reaction conditions were pH 7.2, 37°C, and 1 mM substrate.

**Substrate (No. of carbons in the chain)**	**Activity (U/mg)**
***Lr*Lyp**	
*p*NP-acetate (C2)	0.58 ± 0.05
*p*NP-butyrate (C4)	0.81 ± 0.11
*p*NP-caprylic (C8)	0.25 ± 0.01
*p*NP-laurate (C12)	0.42 ± 0.02
*p*NP-myristate (C14)	0.16 ± 0.02
*p*NP-palmitate (C16)	0.08 ± 0.01
*p*NP-sterate (C18)	0.04 ± 0.01
***Bl*Lyp**	
*p*NP-laurate (C12)	(136 ± 0.7) × 10^−4^

### Stability Over the Time for *Lr*Lyp

**FIGURE 6:**
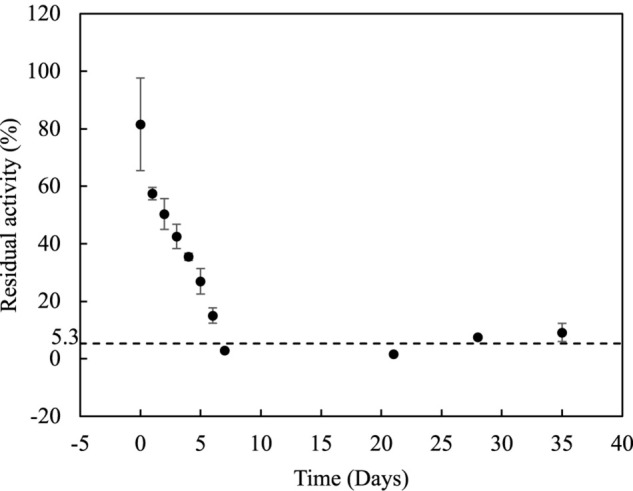
Stability of *Lr*Lyp along the time.

Among the large chain esters, *p*NP-laurate (C12) has shown highest activity (52% comparing with the best substrate *p*NP-butyrate) and it was used for further studies of stability for *Lr*Lyp over time and determination of kinetic constants. Incubating *Lr*Lyp at 37°C, the activity dropped to an average of 5.3% after the 7th day and remained so until more than 35 days (Figure 6).

### Kinetic Constants

**FIGURE 7:**
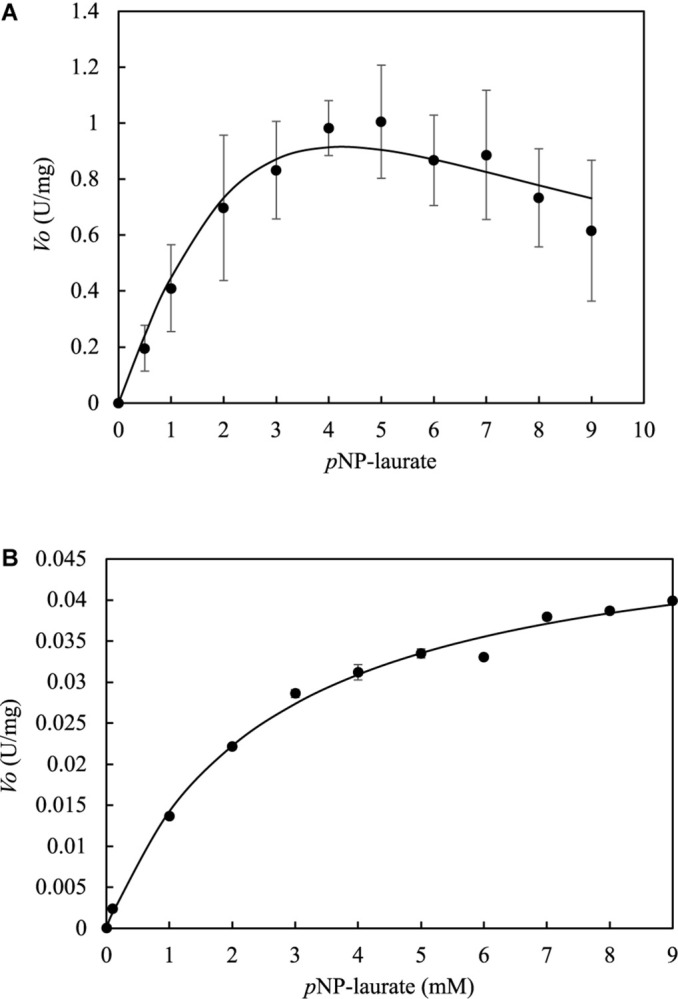
Determination of kinetic constants at pH 7 and 37°C for *Lr*Lyp and *Bl*Lyp by no-linear regression. Error bars represent the standard deviation. *Lr*Lyp **(A)** shows substrate inhibition kinetics, while *Bl*Lyp **(B)** shows a typical Michaelis Menten kinetics. Error bars denote plus/minus one standard deviation.

*Lr*Lyp kinetics does not follow a typical Michaelis-Menten model (Figure 7A). It seems that *Lr*Lyp reaches a saturation point at 5mM of substrate, and at higher substrate concentrations the reaction rate decreases. Which suggest that *Lr*Lyp suffer substrate inhibition at substrate concentrations higher than 5 mM. Therefore, the data were adjusted to the substrate inhibition model to obtain the kinetic constants (Table 2). The coefficient of determination, *R*^2^, of the model was 96%. On the other hand, for *Bl*Lyp Figure 7B shows a typical Michaelis-Menten behavior. The determined apparent kinetic constants are reported in Table 2, and the coefficient of determination was 99%.

**TABLE 2:** Apparent kinetic constants.

**Enzyme**	**$V_{\max}^{\mathrm{app}}$ (U/mg)**	**$K_{M}^{\mathrm{app}}$ (mM)**	**$K_{I}^{\mathrm{app}}$ (mM)**	**$K_{\mathrm{cat}}^{\mathrm{app}}$ (s^–1^)**	**$K_{\mathrm{cat}}^{\mathrm{app}}/K_{M}^{\mathrm{app}}$ (s^–1^/mM)**
*Lr*Lyp	5.8	11.4	1.57	2.6	0.22
*Bl*Lyp	0.05	2.56	–	0.04	0.016

### Multimerization and Aggregation

**FIGURE 8:**
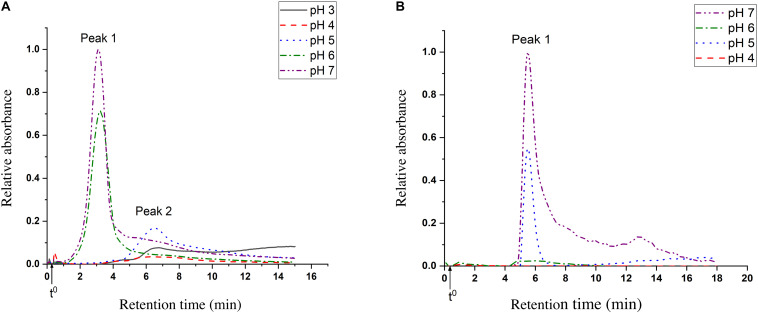
AF4 – UV fractograms of the proteins at different pH. **(A)** *Bl*Lyp, the injected mass is 0.088 mg and the void time t^0^ 17 s. **(B)** *Lr*Lyp, the injected mass is 0.12 mg and the void time (t^0^) is 22 s.

Multimerization and aggregation studies of both *Lr*Lyp and *Bl*Lyp were performed by asymmetric flow field-flow fractionation (AF4). Figure 8A shows the UV – fractograms for *Bl*Lyp at different pHs where two main peaks were identified and characterized respect to their hydrodynamic r_h_ and molar mass. For pH 7, peak 2 absorbs 10 times less than peak 1 (considering peak height). At pH 6 the concentration of peak 1 decrease and peak 2 absorb <10% than peak 1. In pH 5 only peak 2 is present. pH 4 does not show any notable peak, while at pH 3 peak 1 is missing and peak 2 present a tail of other compounds that were not separated. The UV – fractograms for *Lr*Lyp are plotted in Figure 8B, where the hydrodynamic r_h_ for only the first peak was measured. At pH 7 we have the highest absorption for this peak, followed for other peaks that were not completely separated, for pH 6 this peak absorbs <10% compared with pH 7. This peak is prominent at pH 5 again and it disappears completely at pH 4.

For the molecular weight calculations, it is necessary to have both, MALS and UV fractograms. But, due to the MALS signal was not sufficient for *Lr*Lyp, the molecular weight was only calculated for *Bl*Lyp. A characteristic plot for this is shown in Figure 8, where the molecular weight is plotted in the y axis. The resulting molecular weight is the average of the top of the peak (from 2.6 to 3.7 min) and is reported in Table 3 together with the hydrodynamic radii for peak 1 and 2 in Figures 8A,B.

**TABLE 3:** Multimerization analysis.

**Sample**	**Mw (kDa)**	**rh (nm)**	**Multimer form**
*Bl*Lyp – peak 1	53	4	Monomer
*Bl*Lyp – peak 2	n.d	8	Multimer
*Lr*Lyp – peak 1	n.d	3	Monomer?

The results from Table 3 indicate that the Mw obtained from peak 1 (*Bl*Lyp), 53 kDa, agrees with the theoretical Mw (52.36 kDa), therefore it corresponds to the monomeric form with a r_h_ higher than BSA (3.3 nm). The Mw for peak 2 was not calculated because the LS signal-to-noise ratio was insufficient. Figure 8 suggests that *Bl*Lyp is predominantly monomeric at pHs 6 and 7, while at pH 5 it forms multimers. At lower pH no significant peaks were observed, suggesting that the enzyme precipitate or is degraded and lost from the analysis. On the other hand, the r_h_ calculated for *Lr*Lyp suggest that the molecule appears somewhat smaller than BSA (Magnusson et al., 2012) (r_h_ = 3.9 nm and Mw = 66.4 kDa), therefore it could correspond to the monomeric form. If that is the case, then at pHs 5 and 7 the main form would be monomeric, while at the other pH neither multimer nor monomer forms were identified.

### Molecular Modeling of *Lr*Lyp

The search of homologous crystalized proteins in the Protein Data Bank (PDB) via Blast, has given no significant homologous for both enzymes. However, a computational model for *Lr*Lyp was possible to obtain using remote homology techniques with Phyre2 (Kelley et al., 2015). This initial model was validated with ProSa-web (Wiederstein and Sippl, 2007) giving a Z-score of −3.56. This score was improved to −5.21 by refining the initial model by short molecular dynamic simulations (500 ps) with YASARA (Krieger and Vriend, 2014). The refinement has shown improvements in the whole structure, especially in the region of the C-terminal (Supplementary Information S3).

**FIGURE 9:**
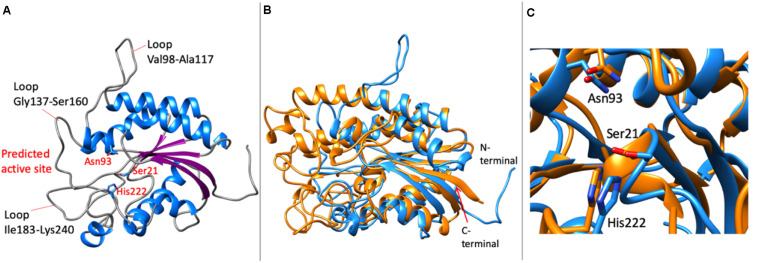
Molecular model of *Lr*Lyp. **(A)** Overall model showing the loops predicted surrounding the catalytic site and putative catalytic amino acids. **(B)** Superimposition of the molecular model (in blue) with the crystallographic structure (in orange) of an esterase from *Streptomyces scabies* (PDB code: 1ESC). **(C)** Detail of the conserved catalytic amino acids predicted by superimposition of the crystallographic structure of the esterase from *S. scabies* and the model of *Lr*Lyp. The catalytic amino acids in the structure 1ESC are Asn106, Ser14, and His283.

The molecular model of *Lr*Lyp resulted in a single domain protein with α/β-hydrolase fold, typical in lipases, esterases and other hydrolases (Figure 9A). The β-sheet is formed by five parallel β-strands and seven α-helixes, in addition, 2_10_ helixes were predicted. In the active site region, long loops are predicted, Val98-Ala117, Gly147-Ser160, Ile183-Lys240 among the longest. These loops can be unmodeled regions due to the lack of crystallographic homologous available in the PDB. On the other hand these loops also can be flexible regions that affect the selectivity of substrates as is discussed in the next section. Despite of the difficulties to model the active site region, interestingly, superimposing the model with the no homologous crystallographic structure (PDB code: 1ESC) of a esterase from *Streptomyces scabies* (Figure 9B), it was found that the catalytic amino acids are well conserved. Thus, Ser21, His222, and Asn93 were predicted as potential catalytic amino acids in *Lr*Lyp (Figure 9C).

## Discussion

Both *Lactobacillus rhamnosus* GG and *Bifidobacterium longum* NCC 2705 are among of the most studied human probiotic strains. Despite that, the molecular bases of many mechanisms involved in the probiotic – host interactions are not clear yet. Probiotic proteins excreted to the extracellular medium could have effect in the interaction with the host. Thus, putative lipolytic enzymes (denoted as Lyp in this work) annotated in the genomes of *L. rhamnosus* GG and *B. longum* have been selected due to the presence of a signal peptide in the N-terminal, which indicates that these proteins are excreted and could have activity on host lipids, fat ingested by the host and even on other metabolites and drugs containing acyl ester bonds.

Based on amino acid sequences analysis, *Lr*Lyp and *Bl*Lyp belong to the lipolytic enzymes families II and IV, respectively. *Lr*Lyp has the GDSL motif, which define the family II. This family differs from the others because contain the amino acid sequence motif GDSL (block I) instead of the typical GXSXG, located in the active site, where S is the catalytic serine (Akoh et al., 2004). There are additional three conserved blocks that characterize this family. Blocks II and III contain conserved glycine (G) and asparagine (N), respectively; both serve as proton donors to the oxyanion hole (Kovacic et al., 2019). Block V contains the catalytic base, histidine (H), which deprotonates the hydroxyl group of the catalytic serine located in the block I, increasing its nucleophilicity (Figure 2). This family is subdivided in two clades, where clade II differs from I because it has an additional motif named block IIIa, no directly involved in catalysis (Leščić Ašler et al., 2010). Because *Lr*Lyp lacks block IIIa, it is subclassified in clade I (Figure 2). Enzymes of this family usually have a broad range of different substrates (Leščić Ašler et al., 2010).

*Bl*Lyp contains the characteristic family IV motif, GDSAGG. This family have four conserved blocks (Arpigny and Jaeger, 1999). The block I contains the consensus sequence HGGG, involved in stabilization of oxyanion hole through hydrogen bonds. Block II contains the catalytic serine (S), while the block IV the catalytic base, histidine (H). Interestingly, the proton donor predicted is a cysteine (C) in *Bl*Lyp, instead of the conserved aspartate (D) (Figure 3). The role of this unusual C will be clarified by mutational analysis in conjunction with structural studies. *Bl*Lyp also contains the sequence YRLA, conserved in the family IV and suggested as block V (Kim et al., 2015). Remarkably, this family groups enzymes with similarity to the mammalian hormone-sensitive lipases (HSL). Nevertheless, the substrate specificity in the bacterial HSL-family differs significantly. Human HSL can hydrolyze a broad range of substrates, both short esters as well as water insoluble substrates, however, bacterial HSL have shown activity on esters of short chain fatty acids (Chahinian et al., 2005).

Despite the classification based on amino acid sequences, these families are still poorly characterized. The experimental results have shown activity on a number of *p*NP-esters, with preference on esters of short chain fatty acids in the case of *Lr*Lyp (Figure 5), which is consistent with the gene annotation. The highest activity was on *p*NP-butyrate; indeed, butyrate and other fatty acids are commonly found in esterified form in plant oils and animal fat. For instance, bovine milk contains a broad variety of both short and large chain fatty acids esterified with glycerol in specific positions (Lindmark Månsson, 2008). The activities reported in this work are significantly lower comparing with the activities of other bacterial lipolytic enzymes. Therefore, it would be interesting to study the activity of *Lr*Lyp and *Bl*Lyp using natural substrates. In fact, the natural substrate and biological function of these novel enzymes remains unknown.

The multimerization study by AF4 indicated that *Lr*Lyp is monomer at pHs 5 and 7, while *Bl*Lyp is monomer at pHs 6 and 7, which suggests that these enzymes are active in monomeric form. Regarding to the kinetic studies, it was difficult to determine accurate kinetic constants for *Lr*Lyp due to the insolubility of the substrates in high concentrations, however the kinetic curve for the substrate *p*NP-laurate shows substrate inhibition when de concentration of the substrate is higher than 5 mM. In the case of *Bl*Lyp, the kinetic constant obeys a Michaelis-Menten behavior for *p*NP-laurate. Comparing the *K*_*M*_ obtained from both enzymes, *Lr*Lyp shows the highest substrate affinity respect to *p*NP-laurate.

On the other hand, the molecular model of *Lr*Lyp obtained has allowed the prediction of catalytic amino acids. Interestingly, the superimposition of the molecular model with the crystallographic structure of a no homologous esterase from *Streptomyces scabies* (Wei et al., 1995) has shown highly conserved catalytic amino acids consistent with the catalytic mechanism suggested for this type of esterases. It is also suggested that the loops surrounding the active site can adopt open and close conformations in lipolytic enzymes, determining the substrate specificity. Thus, the loops predicted in the modeled *Lr*Lyp enzyme could be flexible structures that affect the interactions with the substrates. Crystallographic studies of *Lr*Lyp together with mutational analysis will support to understand the mechanism of reaction of this enzyme.

## Conclusion

It was found that the putative extracellular acyl-hydrolase/lipase from the human probiotic bacteria *Lactobacillus rhamnosus* and *Bifidobacterium longum* have activity on *p*NP-esters. *L. rhamnosus* enzyme has shown preference for *p*NP-esters of short chain fatty acids, and its optimal activity is near to the pH 7 and 37°C. The bioinformatic analysis and computational model has allowed the prediction of catalytic amino acids for the *Lactobacillus rhamnosus* enzyme and suggests that large loops surrounding the active site could determine the substrate specificity. *B. longum* enzyme has been tested on *p*NP-laurate, showing activity at pH 7 and human physiological temperature. On the other hand, both enzymes are active in their monomeric form at pH 7.

## Data Availability Statement

All datasets generated for this study are included in the article/Supplementary Material.

## Author Contributions

JL-P designed the study, did the bioinformatic analysis and modeling, and wrote and corrected the manuscript. LN designed the study, analyzed the data, and corrected the manuscript. PM performed the experiments related to protein purification and kinetic, as well as wrote the first draft of the manuscript. A-SB performed the experiments about multimerization/aggregation analysis. BP produced and purified protein and did the DSF analysis. JP and AH participated in the revision of the manuscript. All authors contributed to the article and approved the submitted version.

## Conflict of Interest

The authors declare that the research was conducted in the absence of any commercial or financial relationships that could be construed as a potential conflict of interest.

## Footnotes

1 www.ebi.ac.uk/Tools/msa/clustalo/
