# Supplementary material for: First Evidence of Acyl-Hydrolase/Lipase Activity From Human Probiotic Bacteria: Lactobacillus rhamnosus GG and Bifidobacterium longum NCC 2705
Source: Front Microbiol. 2020 Jul 24;11:1534. doi: 10.3389/fmicb.2020.01534 (PMC7393678; doi:10.3389/fmicb.2020.01534)
Supplement: Supplementary file 1 [file Table_1.docx]

**Supplementary information**

**First evidence of acyl-hydrolase/ lipase activity from human probiotic bacteria: *Lactobacillus rhamnosus* GG and *Biffidobacterium longum* NCC 2705**

Panagiotis Manasian^1,2^, Atma-Sol Bustos^2,3^, Björn Pålsson^1^, Andreas Håkansson^2^, J. Mauricio Peñarrieta^3^, Lars Nilsson^2*^, Javier A. Linares-Pastén^1*^

^1^ Biotechnology, Faculty of Engineering LTH, Lund University, PO Box 117, S-221 00 Lund, Sweden

^2^ Food Technology, Faculty of Engineering LTH, Lund University, PO Box 124, S-221 00 Lund, Sweden

^3^ School of Chemistry, Faculty of Pure and Natural Sciences, Universidad Mayor de San Andrés, PO Box 303, La Paz, Bolivia

*Corresponding authors: javier.linares_pasten@biotek.lu.se; lars.nilsson@food.lth.se

**S1.** Multiple alignment of selected family II lipolytic enzymes

CLUSTAL O(1.2.4)

*Lr*Lyp ---MRTLKNFFISCGVLLLACLLAFGGWQLFGPVSSPSEVKVVANTPKHLQLTALGDSLT 57

Sc1 ---------MRRFRLVGFLSSLVLAAGAALTGAATAQA-----AQPAAADGYVALGDSYS 46

TesA ------------MRALLLSGCLALVLLTQ----------------QAAAQTLLVVGDSIS 32

SrLip ---------MRLSRRAATASALLLTPALALFGASAAVS-----APRIQATDYVALGDSYS 46

EstHE1 MPRFSALKTSVIRCLTAVALCLVFAGAA-----------------ARDAPVLLVLGDSLS 43

EstA --------MIRMALKPLVAACLLASL-------STAPQ-----AAPSPYSTLVVFGDSLS 40

EstP ---MRKAPLLRFTLASLALACSQ-------------AL-----AGPSPYSTLIVFGDSLA 39

EstE --------MASTLRPIRSLMAVAIAL-------AASPA-----MADSAFDQTVFFGDSLT 40

. .*** :

LrLyp YG--------------VGDATNNGGFVGLTKGELE------------------------- 78

Sc1 SG--------------VGAGSY----------------------ISSSGDCKRSTKAHPY 70

TesA AA--------------LGLDTSQ-GWVALLQKRLA------------------------- 52

SrLip SG--------------VGAGSY----------------------DSSSGSCKRSTKSYPA 70

EstHE1 AA--------------YGMPLSR-GWVSLLEQRLR------------------------- 63

EstA DAGQFPDPAGPAG--STSRFTNRVGPTYQNGSGEIFGPTAPMLLGNQLGIAPGDLAASTS 98

EstP DAGQFPDLVGGTP--G-ARFTN-------RDADGNFAPVSPMILGGRLGVAPGDLNPSTS 89

EstE DSGYYNPLLPAASRAVTGKFTTNPGWVWAEYVGDHFGTNAAPN----------------- 83

. .

*Lr*Lyp --ATGQYQVTTKNYGVSGNTSGQILTRVNKQPK--------------------------I 110

Sc1 LWAAAHSPSTFDFTACSGARTGDVLSGQLGPLS--------------------------- 103

TesA --D-EGYDYRVVNASISGDTSAGGLARLPALL---------------------------- 81

SrLip LWAASHTGTRFNFTACSGARTGDVLAKQLTPVN--------------------------- 103

EstHE1 --D-ANRPWRVVNASISGDTTSGALKRLPKLL---------------------------- 92

EstA PVNAQQGIADGNNWAVGGYRTDQIYDSITAANGSLIERDN----TLLRSRDGYLVDRARQ 154

EstP V----GIQPDGNNWAVGGYTTQQILDSITTTSETVIPPGNPNAGLVLRERPGYLA----N 141

EstE -----GNGQTGDNYAAGGARIQASSVSALGAAPS-----------VTSQVNTYLA---AN 124

. .*

*Lr*Lyp RADLKRANIITVTAGGNDLMHVLQKHFLTLSEKQVTAGSVA---------FQKRLATLLT 161

Sc1 ----SGTGLVSISIGGNDAGFADTMTTCVLQSESSCLSRIATAEAYVDSTLPGKLDGVYS 159

TesA --AEEKPALVVIELGGNDGLRGMAPAQ-----------------------LQQNLASMAQ 116

SrLip ----SGTDLVSITIGGNDAGFADTMTTCNLQGESACLARIAKARAYIQQTLPAQLDQVYD 159

EstHE1 --ELHAPEVVIIELGGNDGLQGKPLDT-----------------------IASNLQGLIS 127

EstA GLGADPNALYYITGGGNDFLQGRILNDVQAQ---Q---------------AAGRLVDSVQ 196

EstP GLRADPNALYYLTGGGNDFLQGLVNSPADAV---A---------------AGARLAASAQ 183

EstE GGQANPNALYTVWGGANDLLAAATAPAQAQTIIGS---------------AVTAQVGAVG 169

: : *.**

*Lr*Lyp TIRKENPTAPIY----VFGIYNPFYVYFPKMTA-MTNSVKAWNQATQKTLQQFDR-TYYV 215

Sc1 AISDKAPNAHVV-----VIGYPRFYKLGTTCI-G------------LSETKRTAINKASD 201

TesA KARAEGAKVLLLG-IQLPPNYGPRYIEAFSRVY-GAVA---------------------- 152

SrLip AIDSRAPAAQVV-----VLGYPRFYKLGGSCAVG------------LSEKSRAAINAAAD 202

EstHE1 VVRGAGAQPALVG-MRIPPNYGRYYTGEFERLY-EQIA---------------------- 163

EstA ALQQAGARYIVVWLLPDLGLTPATFGGPLQPFA--SQLSGTFNAELTAQLSQAGANVIPL 254

EstP ALQQGGARYIMVWLLPDLGQTPNFSGTPQQNPL--SLLSAAFNQSLISQLGQIDAQIIPL 241

EstE ALQAAGARYVMVPTIPDVGITPRFRAGGAAAMAQGTAAATAYNTALFNGLQSAGLRVIPV 229

:

*Lr*Lyp DIDSVLSNGETAANKAS-------AKEALKEATSGDGNPL------------IFSQDHFH 256

Sc1 HLNTVLAQRAAA-HGFTFGDVRT----TFTGHELCSGSPW------LHSVNWLNIGESYH 250

TesA -------------AQEK-------TALVPFFLEGVGGVQG------------MMQADGIH 180

SrLip DINAVTAKRAAD-HGFAFGDVNT----TFAGHELCSGAPW------LHS-VTLPVENSYH 250

EstHE1 -------------EREE-------VPLLRFGLEGLASARG------------MMQEDGIH 191

EstA NIPLLLKEGMANPASFGLAADQN-LIGTCFSGNGCTMNPTYGINGSTPDPSKLLFNDSVH 313

EstP NIPLLLSEALASPSQFGLASDQN-LVGTCYSGDSCVENPVYGINGTTPDPTKLLFNDSVH 300

EstE DTFHILQEVVADPGIYGFSNVTGTACNPALALPACNPTSL----VAANAPNTYVFADGIH 285

: *

*Lr*Lyp PNNAGYGQMTKQLMKQMKATKKAWE----------------------------------- 281

Sc1 PTAAGQSGGYLPVLNGAA------------------------------------------ 268

TesA PALAAQPRLLENVWPTLKPLL--------------------------------------- 201

SrLip PTANGQSKGYLPVLNSAT------------------------------------------ 268

EstHE1 PAPEAQARMLDSLWPDLNAELLPLEQGAPLDH---------------------------- 223

EstA PTITGQRLIADYTYSLLS---APWELTLLPEMAHGTLRAYQDELRSQWQADWENWQNVGQ 370

EstP PTIAGQQLIADYAYSILA---APWELTLLPEMAHASLRAHQDELRNQWQTP---WQAVGQ 354

EstE PTTATHQILGQYAISLLE---APRLQQVLTRSAQAGGRARADQV--AWHLDGKPEADGLR 340

*

*Lr*Lyp ------------------------------------------------------------ 281

Sc1 ------------------------------------------------------------ 268

TesA ------------------------------------------------------------ 201

SrLip ------------------------------------------------------------ 268

EstHE1 ------------------------------------------------------------ 223

EstA WRGFVGGGGQRLDFDSQDSAASGDGNGYNLTLGGSYRIDEAWRAGVAAGFYRQKLEAGAK 430

EstP WQAFVASGAQDLDFDGQHSAASGDGRGYNLTVGGSYRLNDAWRLGLAGGANRQKLEAGEQ 414

EstE WWGSVRGDIQRYDDAD-----LYDGMAPAGLFGVDWTA-GDLVFGGFAGFGRMDADFGNR 394

*Lr*Lyp ------------------------------------------------------------ 281

Sc1 ------------------------------------------------------------ 268

TesA ------------------------------------------------------------ 201

SrLip ------------------------------------------------------------ 268

EstHE1 ------------------------------------------------------------ 223

EstA DSDYRMNSYMASAFVQYQENRWWADAALTGGYLDYDDLKRKFALGGGERSEKGDTNGHLW 490

EstP DSDYKLNSYMASAFAQYRQDRWWADAALTAGHLDYSDLKRTFALGVNDRSEKGDTDGEAW 474

EstE NGSFKQDDTTLGGFVGWYTGPVWVNAQVSYSWLSYD-VDREVQLGPATRVHSGAPDGSNL 453

*Lr*Lyp ------------------------------------------------------------ 281

Sc1 ------------------------------------------------------------ 268

TesA ------------------------------------------------------------ 201

SrLip ------------------------------------------------------------ 268

EstHE1 ------------------------------------------------------------ 223

EstA AFSARLGYDIAQQADSPWHLSPFVSADYARVEVDGYSEKGASATALDYDDQKRSSKRLGA 550

EstP AMSGRLGYNLAAD-TSNWQLAPFISADYARVKVDGYDEKSGRSTALGFDDQERTSRRLGV 533

EstE TAAVNAGYSLG---EGNVKYGPVVGLTWQKLKLDGYTESNASSTALGYADQDIDSLVGRI 510

*LrLyp* ------------------------------------------------------------ 281

Sc1 ------------------------------------------------------------ 268

TesA ------------------------------------------------------------ 201

SrLip ------------------------------------------------------------ 268

EstHE1 ------------------------------------------------------------ 223

EstA GLQGKYAFGSDTQLFAEYAHEREYEDDTQDLTMSLNSLPGN-RFTLEGYTPQDHLNRVSL 609

EstP GLLGSVQVLPSTRLFAEVAQEHEFEDDEQDVTMHLTSLPAN-DFTLTGYTPHSDLTRASL 592

EstE GFQVRLDGAP-VKPYLQATYDHEFKDGTEASA-WLQSMPEVGMYTVPGQNFDRNYATVVL 568

*Lr*Lyp ---------------------------------------- 281

Sc1 ---------------------------------------- 268

TesA ---------------------------------------- 201

SrLip ---------------------------------------- 268

EstHE1 ---------------------------------------- 223

EstA GFSQKLA---PELSLRGGYNWRKGEDDTQQSVSLALSLDF 646

EstP GVSHELV---AGVHLRGNYNWRKSDELTQQGISVGVSVDF 629

EstE GARTGIWGLQSNIGLSTTTAQRSARD---ATVFVNFSGNF 605

**S2.** Multiple alignment of selected family IV lipolytic enzymes

LUSTAL O(1.2.4)

*Bl*Lyp MARWVTRSVPATACTSTCPATSTSRSSRPAWNTSPPDNRRVSRLIRHTGKGLSGSFPFCS 60

Est3K ------------------------------------------------------------ 0

Pseudomonas ------------------------------------------------------------ 0

Moraxella -----MPILPVPALNALLT------KTIKTIKTGAAKNAHQHHVLHHTLKGLD------- 42

2HM7 ------------------------------------------------------------ 0

5L2P ------------------------------------------------------------ 0

3WJ1 ------------------------------------------------------------ 0

*Bl*Lyp CRSWAKVGVFALPNPPLK---------GPVVMPINEALFAAMKAASYIKPN-AGKSYKLQ 110

Est3K ----------------------------------------------------------MQ 2

Pseudomonas -------------------------------MPLD-KQIAAVLQQFSELPAPDFSQLDAA 28

Moraxella ----------NLPAPVLERINRRLKASTAEQYPLADAHLRLILAISNKLKRPLA-IDKLP 91

2HM7 -------------------------------MPLD-PVIQQVLDQLNRMPAPDYKHLSAQ 28

5L2P -------------------------------MPLD-PEVRNFLQVYYKANIIDFTKYQFQ 28

3WJ1 -------------------------------MPLD-PRIKKLLESGFVVPIGKA---SVD 25

*Bl*Lyp RVAEE--------LIAKQA--------------PANPKCRVEDAFAPMADGYAVPLRVFT 148

Est3K RFNQKLAWMPRFRIRNRVTPRVIQALLRSSQMVAGNKLLK-HGLQAESRRVGSVPVRIIR 61

Pseudomonas QYRQFCDN---------LLP----------A-IPGDPMIEVR-NLRVAAAAGELDARLYR 67

Moraxella KLRQKFGTD----AVSLQAPSVWQQNADASG-STENAVSWQD-KTIANADGGDMTVRCYQ 145

2HM7 QFRSQ-QS---------LFP----------P-VKKEPVAEVR-EFDMDLPGRTLKVRMYR 66

5L2P EIRQKVNE--------LLAK----------A-VPKDPVGETR-DMKIKLEDYELPIRIYS 68

3WJ1 EVRKIFRQ---------LAS----------A-APKAEVRKVE-DIKIPGSETSINARVYF 64

. . : *

*Bl*Lyp PLVAYGAPLPEALEESSANGTPVASAISAILPAVLPKVLPKILIKESTSSGNADGISGNA 208

Est3K ------------------------------------------------------------ 61

Pseudomonas P---------------------------------------------------------LE 70

Moraxella K---------------------------------------------------------ST 148

2HM7 P---------------------------------------------------------EG 69

5L2P P---------------------------------------------------------IK 71

3WJ1 P---------------------------------------------------------KA 67

*Bl*Lyp NTELPSVTPRGTILFFHGGGWTTGGINLYTQACAHMAVRLQRRVISVEYRLAPEYRFPTA 268

Est3K ----PKGKAKGVVLDIHGGGWVIGNAQMNDDLNVAMVNACEVAVVSVDYRLAVNTPVEGI 117

Pseudomonas ------EDNLPLLVFFHGGGFVMGNLDTHDNLCRSLASQTEAVVVSVAYRLAPENHFPAA 124

Moraxella QNSERKSTDEAAMLFFHGGGFCIGDIDTHHEFCHTVCAQTGWAVVSVDYRMAPEYPAPTA 208

2HM7 -----VEPPYPALVYYHGGSWVVGDLETHDPVCRVLAKDGRAVVFSVDYRLAPEHKFPAA 124

5L2P ------RTNNGLVMHFHGGAWILGSIETEDAISRILSNSCECTVISVDYRLAPEYKFPTA 125

3WJ1 ------KGPYGVLVYLHGGGFVIGDVESYDPLCRAITNACNCVVVSVDYRLAPEYKFPSA 121

:: ***.: *. : : *.** **:* :

*Bl*Lyp VEDCYEVARQLFAGELPISGVGGSVDVDHPQSAAPTAPAGGGDISATIPAPDPDSIVLFG 328

Est3K LEDCLATARWLLADC-------------------------------EEFA--GLPVIVVG 144

Pseudomonas PLDCYAATCWLVEHA-------------------------------AELGVDGRRLALAG 153

Moraxella LKDCLAAYAWLAEHS-------------------------------QSLGASPSRIVLSG 237

2HM7 VEDAYDALQWIAERA-------------------------------ADFHLDPARIAVGG 153

5L2P VYDCFNAIVWARDNA-------------------------------GELGIDKDKIATFG 154

3WJ1 VIDSFDATNWIYNNL-------------------------------DKFDG-EMGIAIAG 149

*. . : *

*Bl*Lyp DSAGGNLAAAVSLMARD---------------------------RGEFMPRTQMLLYPVV 361

Est3K ESAGGHLAAATLLALKQSPEL-------------------------LARVSGAVLYYGVY 179

Pseudomonas DSAGGNLALAVSRLAAQ---------------------------RQGPKISYQCLFYPVT 186

Moraxella DSAGGCLAALVAQQVIKPIDALWQDNNQAPAADKKVNDTFKNSLADLPRPLAQLPLYPVT 297

2HM7 DSAGGNLAAVTSILAKE---------------------------RGGPALAFQLLIYPST 186

5L2P ISAGGNLVAATSLLARD----------------------------NKLKLTAQVPVVPFV 186

3WJ1 DSAGGNLAAVVALLSK-----------------------------GKLDLKYQILIYPAV 180

**** *. .

*Bl*Lyp GNDYNPETSPFESVRTNGT-DYILTAQDMAD-YIDMYR-SSVADLTNPYFAPLTAHDLSN 418

Est3K DLT------GTPSVRTAGRETLLLDGPGMVEALRLLTPGLSDEQRRQPPLSP-LYGDLAG 232

Pseudomonas DAR-----CDSQSYEEFAE-GYFLTGAMMYW-FWQQYL-QDTGQGDDPLASPLRAETLAD 238

Moraxella DYE-----AEYPSWELYGE-GLLLDHNDAEV-FNSAYT-QHSGLPQSHPLISVMHGDNTQ 349

2HM7 GYDPA---HPPASIEENAE-GYLLTGGMMLW-FRDQYL-NSLEELTHPWFSPVLYPDLSG 240

5L2P YLD-----LASKSMNRYRK-GYFLDINLPVDYGVKMYI-RDEKDLYNPLFSPLIAEDLSN 239

3WJ1 GFD-----SVSRSMIEYSD-GFFLTREHIEW-FGSQYL-RSPADLLDFRFSPIIAQDLSG 232

* :* :

*Bl*Lyp QPRTLVLSAEYCPLRDEDEAYARRLQLVNDNVSCYRIHDGIHGYLLNTSAVGLVATT-YR 477

Est3K LPPALMFVGELDPLLDDTLQMAERWAGSE----VFLLPQAAHGFIHFPTAMSGRVLAYSR 288

Pseudomonas LPPTTLITAEFDPLRDEGEAFALRLQQAGVSVRVQRCEGMIHGFISMAPFVERAAHALSD 298

Moraxella LCPSYIVVAELDILRDEGLAYAELLQKEGVQVQTYTVLGAPHGFINLMSVHQGLGNQTTY 409

2HM7 LPPAYIATAQYDPLRDVGKLYAEALNKAGVKVEIENFEDLIHGFAQFYSLSPGATKALVR 300

5L2P LPQAIVVTAEYDPLRDQGEAYAYRLMESGVPTLSFRVNGNVHAFLGSPRTSRQVTV---M 296

3WJ1 LPPALIITAEYDPLRDQGEAYANRLLQAGVPVTSVRFNNVIHGFLSFFPLIDQGKDAIGL 292

: : .: * * * *.:

*Bl*Lyp IIEHFLDGTPLEPAPGTGTTANAPEGGDAWQDVLGTD 514

Est3K E--WITGRL-----RSVG------------------- 299

Pseudomonas A----AADLR-RALN---------------------- 308

Moraxella IINEFACLVQ-NLLTSEGDKPNLRA------------ 433

2HM7 I----AEKLR-DALA---------------------- 310

5L2P I----GALLK-DIFK---------------------- 306

3WJ1 I----GSVLR-RTFYDKS------------------- 305

S3. Validation of the computational model of Lb*Lyp*


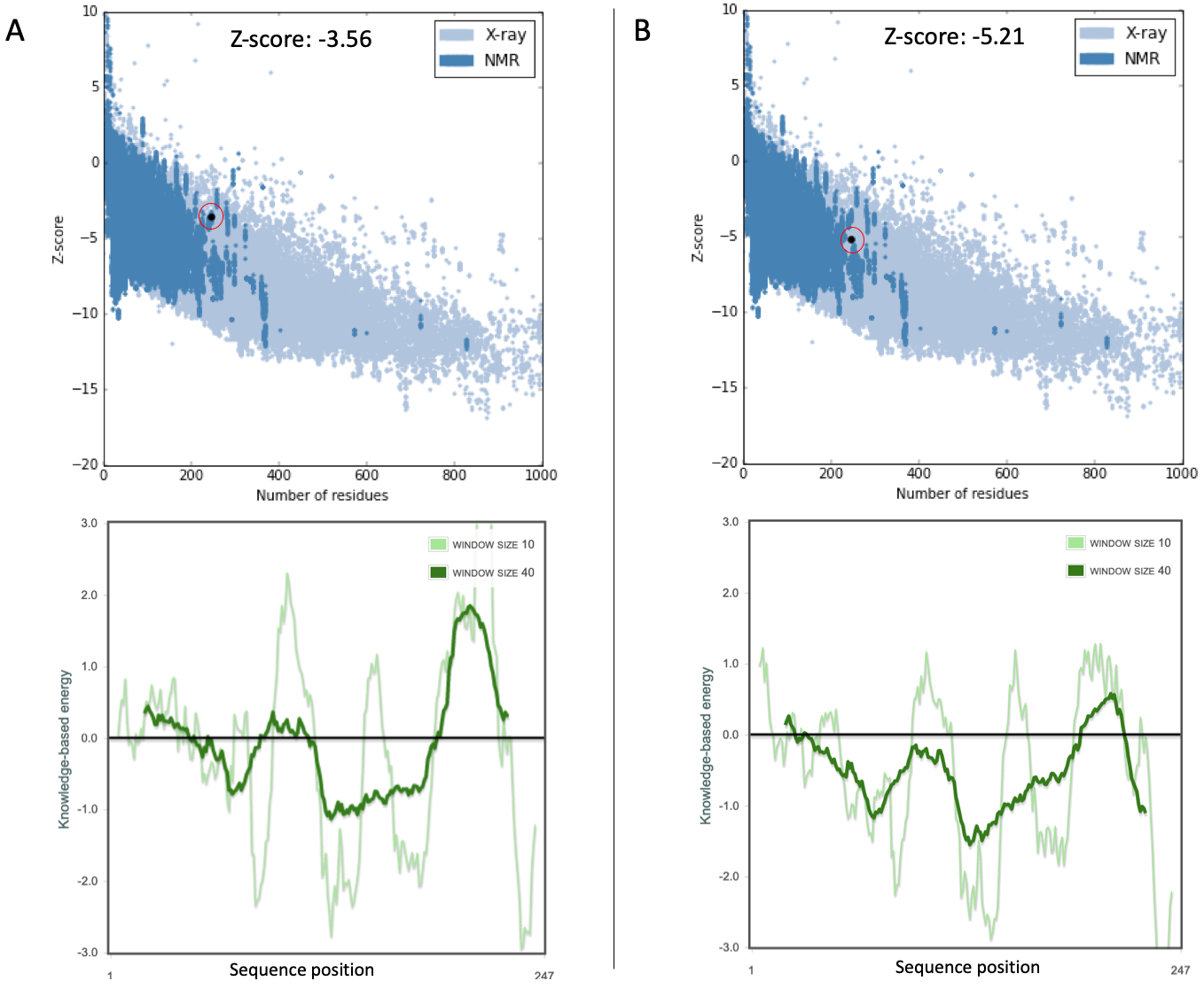


**S3.** Validation of the computational model of *Lb*Lip using ProSa-web (Wiederstein and Sippl, 2007). (A) Initial model obtained using remote homology aproach with Phire2 (Kelley et al., 2015). (B) Model refined by molecular dynamic simulations using YASARA (Krieger and Vriend, 2014). The top plots shows the overal model quality (Z-scores) and the botton plots shows the local model quality.

**References**

WIEDERSTEIN, M. & SIPPL, M. J. 2007. ProSA-web: interactive web service for the recognition of errors in three-dimensional structures of proteins. *Nucleic acids research,* 35**,** W407-W410.

KELLEY, L. A., MEZULIS, S., YATES, C. M., WASS, M. N. & STERNBERG, M. J. 2015. The Phyre2 web portal for protein modeling, prediction and analysis. *Nature protocols,* 10**,** 845.

KRIEGER, E., DARDEN, T., NABUURS, S. B., FINKELSTEIN, A. & VRIEND, G. 2004. Making optimal use of empirical energy functions: force‐field parameterization in crystal space. *Proteins: Structure, Function, and Bioinformatics,* 57**,** 678-683.

Kelley, L.A., Mezulis, S., Yates, C.M., Wass, M.N., and Sternberg, M.J. (2015). The Phyre2 web portal for protein modeling, prediction and analysis. *Nature protocols* 10**,** 845.

Krieger, E., and Vriend, G. (2014). YASARA View—molecular graphics for all devices—from smartphones to workstations. *Bioinformatics* 30**,** 2981-2982.

Wiederstein, M., and Sippl, M.J. (2007). ProSA-web: interactive web service for the recognition of errors in three-dimensional structures of proteins. *Nucleic acids research* 35**,** W407-W410.
